# Supplementary material for: Genetic diversity affects ecosystem functions across trophic levels as much as species diversity, but in an opposite direction
Source: eLife. 2025 Mar 20;13:RP100041. doi: 10.7554/eLife.100041 (PMC11925449; doi:10.7554/eLife.100041)
Supplement: Supplementary file 1. — A Wald chi-square test is used to test the significance of each fixed effect. [file elife-100041-supp1.docx]

# SUPPLEMENTARY FILE 1

**Table S1.** ANOVA table for the linear mixed model testing whether the relationships between biodiversity and ecosystem functions measured in a riverine trophic chain differ between the biodiversity facets (species or genetic diversity), the types of BEF (*within-* or *between-trophic levels*) and the trophic levels at which BEFs are estimated (primary producers, primary consumers or secondary consumers). A Wald chi-quare test is used to test the significance of each fixed effect.

|  | Degree of freedom | Chisq-value | P-value |
| --- | --- | --- | --- |
| (Intercept) | 1 | 1.453 | 0.228 |
| Biodiversity facet | 1 | 1.293 | 0.255 |
| Type of BEF | 1 | 7.498 | **0.006** |
| Trophic level | 2 | 1.976 | 0.372 |
| Biodiversity facet*Type of BEF | 1 | 7.884 | **0.005** |
| Biodiversity facet*Trophic level | 2 | 3.520 | 0.172 |
| Trophic level*Type of BEF | 2 | 2.901 | 0.234 |
| Biodiversity facet*Type of BEF*Trophic level | 1 | 2.994 | 0.224 |
