# Supplementary material for: Genetic diversity affects ecosystem functions across trophic levels as much as species diversity, but in an opposite direction
Source: eLife. 2025 Mar 20;13:RP100041. doi: 10.7554/eLife.100041 (PMC11925449; doi:10.7554/eLife.100041)
Supplement: Supplementary file 2. — 95% confidence intervals are provided together with the estimate of each BEF. BEFs are considered as significant when the 95% CI does not overlap 0. p-Values estimated from t-test are also provided. [file elife-100041-supp2.docx]

**SUPPLEMENTARY MATERIAL 2**

**Table S2 – Estimates of individual effect sizes of BEFs (Zr, n=34) for each ecosystem function, each biodiversity facet (genetic or species diversity) and each type of BEF (within- or between-trophic levels).** 95% confidence intervals are provided together with the estimate of each BEF. BEFs are considered as significant when the 95%CI does not overlap 0. P-values estimated from t-test are also provided.

| **Ecosystem function** | **Predictor** | **Biodiversity facet** | **Type of BEF** | ***Zr*** | **negative 95% CI** | **positive 95% CI** | **P values** |
| --- | --- | --- | --- | --- | --- | --- | --- |
| ***Phoxinus* biomass** | **Fish species diversity** | **Species diversity** | **Whithin-trophic levels** | **-0.529** | **-0.803** | **-0.166** | **0.006** |
| ***Alnus* biomass** | **Tree species diversity** | **Species diversity** | **Whithin-trophic levels** | **-0.447** | **-0.695** | **-0.143** | **0.006** |
| ***Phoxinus* biomass** | ***Alnus* genetic diversity** | **Genetic diversity** | **Between-trophic levels** | **-0.321** | **-0.602** | **-0.020** | **0.045** |
| Litter decomposition | Tree species diversity | Species diversity | Whithin-trophic levels | -0.293 | -0.630 | 0.060 | 0.115 |
| *Gammarus* biomass | Invertebrate species diversity | Species diversity | Whithin-trophic levels | -0.273 | -0.661 | 0.128 | 0.194 |
| Litter decomposition | Fish species diversity | Species diversity | Between-trophic levels | -0.210 | -0.625 | 0.210 | 0.337 |
| Tree biomass | Tree species diversity | Species diversity | Whithin-trophic levels | -0.183 | -0.485 | 0.122 | 0.250 |
| Fish biomass | Invertebrate species diversity | Species diversity | Between-trophic levels | -0.138 | -0.428 | 0.153 | 0.506 |
| *Phoxinus* biomass | Invertebrate species diversity | Species diversity | Between-trophic levels | -0.129 | -0.471 | 0.215 | 0.362 |
| Invertebrate biomass | Invertebrate species diversity | Species diversity | Whithin-trophic levels | -0.051 | -0.381 | 0.279 | 0.766 |
| Fish biomass | *Gammarus* genetic diversity | Genetic diversity | Between-trophic levels | -0.047 | -0.457 | 0.364 | 0.825 |
| *Gammarus* biomass | *Alnus* genetic diversity | Genetic diversity | Between-trophic levels | -0.043 | -0.438 | 0.352 | 0.833 |
| Litter decomposition | Invertebrate species diversity | Species diversity | Between-trophic levels | -0.020 | -0.401 | 0.361 | 0.918 |
| Litter decomposition | *Alnus* genetic diversity | Genetic diversity | Whithin-trophic levels | -0.012 | -0.394 | 0.370 | 0.950 |
| Litter decomposition | *Phoxinus* genetic diversity | Genetic diversity | Between-trophic levels | 0.018 | -0.379 | 0.416 | 0.930 |
| Invertebrate biomass | *Alnus* genetic diversity | Genetic diversity | Between-trophic levels | 0.025 | -0.306 | 0.356 | 0.885 |
| Invertebrate biomass | Tree species diversity | Species diversity | Between-trophic levels | 0.063 | -0.236 | 0.362 | 0.682 |
| Invertebrate biomass | *Phoxinus* genetic diversity | Genetic diversity | Between-trophic levels | 0.085 | -0.260 | 0.429 | 0.634 |
| Fish biomass | Tree species diversity | Species diversity | Between-trophic levels | 0.087 | -0.276 | 0.449 | 0.644 |
| Invertebrate biomass | *Gammarus* genetic diversity | Genetic diversity | Whithin-trophic levels | 0.097 | -0.242 | 0.435 | 0.580 |
| Litter decomposition | *Gammarus* genetic diversity | Genetic diversity | Between-trophic levels | 0.110 | -0.281 | 0.500 | 0.586 |
| *Alnus* biomass | *Alnus* genetic diversity | Genetic diversity | Whithin-trophic levels | 0.128 | -0.179 | 0.433 | 0.422 |
| Invertebrate biomass | Fish species diversity | Species diversity | Between-trophic levels | 0.144 | -0.219 | 0.504 | 0.444 |
| Fish biomass | *Phoxinus* genetic diversity | Genetic diversity | Whithin-trophic levels | 0.159 | -0.260 | 0.575 | 0.464 |
| *Gammarus* biomass | Tree species diversity | Species diversity | Between-trophic levels | 0.161 | -0.198 | 0.516 | 0.388 |
| *Phoxinus* biomass | Tree species diversity | Species diversity | Between-trophic levels | 0.165 | -0.100 | 0.426 | 0.233 |
| *Gammarus* biomass | Fish species diversity | Species diversity | Between-trophic levels | 0.185 | -0.249 | 0.614 | 0.413 |
| Fish biomass | *Alnus* genetic diversity | Genetic diversity | Between-trophic levels | 0.193 | -0.211 | 0.592 | 0.360 |
| *Phoxinus* biomass | *Gammarus* genetic diversity | Genetic diversity | Between-trophic levels | 0.228 | -0.074 | 0.522 | 0.151 |
| *Phoxinus* biomass | *Phoxinus* genetic diversity | Genetic diversity | Whithin-trophic levels | 0.244 | -0.064 | 0.542 | 0.133 |
| Tree biomass | *Alnus* genetic diversity | Genetic diversity | Whithin-trophic levels | 0.280 | -0.062 | 0.609 | 0.121 |
| *Gammarus* biomass | *Gammarus* genetic diversity | Genetic diversity | Whithin-trophic levels | 0.292 | -0.120 | 0.688 | 0.178 |
| Fish biomass | Fish species diversity | Species diversity | Whithin-trophic levels | 0.326 | -0.123 | 0.753 | 0.169 |
| ***Gammarus* biomass** | ***Phoxinus* genetic diversity** | **Genetic diversity** | **Between-trophic levels** | **0.446** | **0.007** | **0.830** | **0.047** |
